# Diagnostic Performance of ANCA Testing Across Three Platforms with and without Coexisting ANA Positivity: Experience from a Tertiary Healthcare Centre in India

**DOI:** 10.31138/mjr.130225.eft

**Published:** 2025-08-20

**Authors:** Neha Rai, Shamshad Ahmad, Saurabh Karmakar, Divendu Bhushan, Pragya Kumar, Ayan Banerjee, Mukunda Kumar, Akash Bansal, Anurag Kumar, Rajiv Ranjan Sinha, Mala Mahto

**Affiliations:** 1 Department of Biochemistry, AIIMS Patna, Patna, Bihar, India;; 2 Department of CFM, AIIMS Patna, Patna, Bihar, India;; 3 Department of Pulmonary Medicine, AIIMS Patna, Patna, Bihar, India;; 4 Department of Emergency Medicine, AIIMS Patna, Patna, Bihar, India;; 5 Department of Biochemistry, LCMCH, Palamu, Jharkhand, India;; 6 Department of Biochemistry, AIIMS Gorakhpur, Uttar Pradesh, India;; 7 Department of Biochemistry, NMCH, Patna, Bihar, India

**Keywords:** ANA interference, IIF, ELISA, pANCA, cANCA, vasculitis

## Abstract

**Background:**

Indirect immunofluorescence (IIF) is a useful diagnostic modality for anti-neutrophilic cytoplasmic antibody (ANCA) detection in ANCA associated with vasculitis and diseases beyond vasculitis. As per latest guidelines, the IIF has been replaced by enzyme-linked immunosorbent assay (ELISA) as first line of screening for ANCA. The study intends to evaluate the performance of IIF and ELISA for ANCA testing and the impact of antinuclear antibody (ANA) positivity on ANCA reporting across these platforms.

**Methods:**

A total of 70 samples, 53 ANA positive and 17 ANA negative, were included in the study. They were tested for ANCA across three different platforms based on IIF and ELISA. An attempt was also made to identify if presence and pattern of ANA affected ANCA reporting across any particular platform.

**Results:**

The impact of ANA positivity on ANCA reporting was done using logistic regression analysis. An evaluation of the sensitivity and specificity of each ANCA testing platform was done considering ELISA as the reference method. Subgroup analyses were conducted on the basis of ANA patterns to further know their impact on the specificity and sensitivity of ANCA tests. The use of a 3-biochip combination for ANCA reporting by IIF resulted in a significant reduction of false positive ANCA due to ANA when a combination of ethanol and formalin fixed granulocytes were used.

**Conclusion:**

There are notable differences in ANCA diagnostics between IIF based on ethanol fixed granulocytes alone and combination of three biochip. However, the addition of Proteinase 3/myeloperoxidase (PR3/MPO) dots to the 3-chip combination did not offer any added advantage.

## INTRODUCTION

Anti-neutrophilic cytoplasmic antibodies (ANCAs) are auto antibodies which target components within granules located in the cytoplasm of neutrophils and monocytes. Testing for ANCA in the clinical laboratory is usually done to aid the diagnosis of ANCA-associated vasculitis (AAV), which usually affects small and medium vessels.^1^ ANCA includes two distinct population of antibodies which bind either to myeloperoxidase (MPO) or Proteinase 3 (PR3) within neutrophilic granules. They are represented as anti-MPO antibodies or perinuclear ANCA (pANCA) and anti-PR3 or cytoplasmic ANCA (cANCA) respectively.^2^ Anti-MPO antibodies are usually present in microscopic polyangiitis (MPA), and anti-PR 3 is generally associated with Wegner’s granulomatosis.

However, ANCAs are also known to be associated with diseases apart from vasculitis like inflammatory bowel disease; mainly, ulcerative colitis, autoimmune hepatitis, infective endocarditis, malignancy, etc., where antigens such as lactoferrin, cathepsin, bactericidal permeability increasing protein (BPI), lysozyme, etc. are involved. ANCA in such cases is referred to as atypical ANCA.^3^

Indirect immunofluorescence (IIF) is one of the most common techniques used for ANCA diagnostics, as it can be used to detect not only ANCA associated with vasculitis, but also ANCAs in diseases beyond vasculitis as stated above.^4^ IIF has been the preferred test since the 1999 consensus statement, with PR3/MPO-ANCA reflex testing.^5^ IIF is performed with ethanol and formalin-fixed human neutrophils as substrate and two main fluorescence patterns are noticed: a granular cytoplasmic pattern (c-ANCA) or a perinuclear pattern (p-ANCA). pANCA pattern reflects presence of anti-myeloperoxidase antibodies (MPO-ANCA) in 90% of the cases, and 10% is PR-3 reactive. cANCA corresponds to anti-proteinase 3 antibodies (PR3-ANCA) in 90% of the cases, and is MPO-reactive in 10% of the cases.^6^ However, the Revised International Consensus (2017) recommends the use of highly specific and good quality mono-antigen targeted assays for primary screening and detection of PR3-ANCA and MPO-ANCA, thereby substituting IIF as the primary testing methodology in AAV.^7^ Immunoassays like enzyme-linked immunosorbent assays (ELISA) make use of purified specific antigens. Immunoassays which detect antibodies against PR3/MPO are believed to be associated with better specificities and positive predictive values in comparison to IIF patterns. It further states that an alternative immunoassay needs to be performed for patients with negative reports despite a high clinical suspicion of vasculitis (for increased sensitivity) or in patients with low antibody levels (for increased specificity). It should be asserted here that the 2017 consensus statement claims that it is not based on evidence-based guidelines or meta-analysis. The authors also accept that these recommendations require further evaluation in the future.^7^ Moreover, ANCA testing yields positive results in diseases beyond vasculitis with an “atypical pattern” routinely detectable only by IIF. In 2020, a revised document on ANCA testing was published as a follow up on a 2017 revised international consensus focusing on ANCA testing in diseases beyond systemic vasculitis.^8^ Although monospecific assays are considered superior in the diagnosis of ANCA, IIF is crucial, as many antigens are yet to be identified. Almost all ANCA targeted antigens are detected by IIF, as it also detects the presence of antibodies for antigens apart from MPO and PR3, which are the only two antigens most commonly included in the standard ANCA monospecific test. Another challenge to consider with ANCA reporting is that the pattern of true pANCA can be difficult to distinguish from the pattern caused by presence of an existing antinuclear antibody (ANA). Hence, checking for ANA in cases of pANCA positivity is imperative to avoid false positives.^9^ Currently, other immunoassays for determining ANCA are available, although not very commonly used.^4^ Interference of ANA may result in false positive ANCA when tested; using IIF can be mitigated to great extent with the newer approaches.^9^ With no definitive protocols established for ANCA testing for disease screening or monitoring in view of multiple challenges, this study tries to explore the suitability of ANCA reporting across three different platforms in cases of coexisting ANA positivity.

## METHODS

### Study design

Prospective.

### Participants

**Figure 1.**
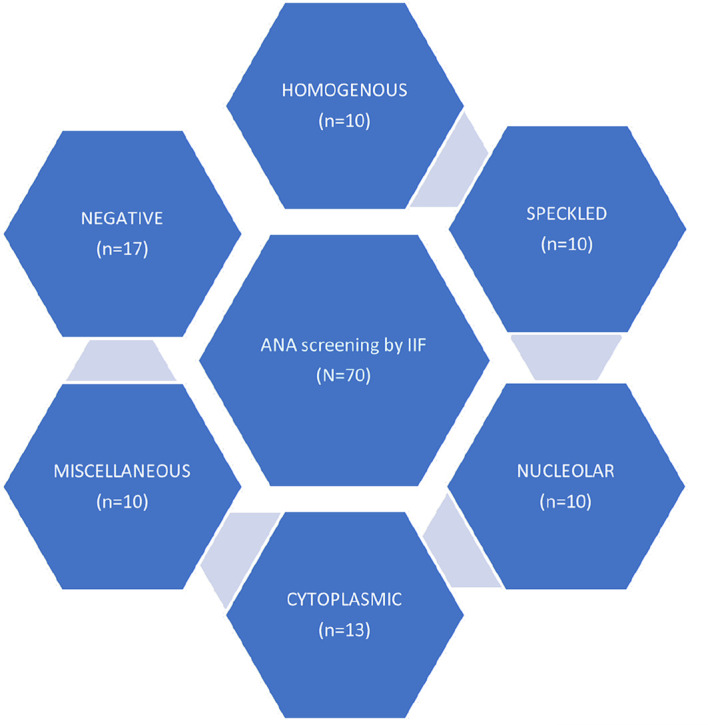
**(above left).** Different ANA subgroups enrolled in the study based on screening by IIF.

A total of 70 blood samples on which ANA screening by IIF was ordered by the treating clinician and received in Central Laboratory (Biochemistry wing) from April 2023 to November 2023 were selected for the study. The basis for inclusion was ANA positivity of intensity two or more with 5 different patterns namely homogenous(n=10), speckled (10), nucleolar (10), cytoplasmic (n=13), and miscellaneous (10). Miscellaneous included patterns other than the four mentioned above. A total of 17 samples negative for ANA by IIF were also included (**Figure 1**).

### Test method

**Figure 2.**
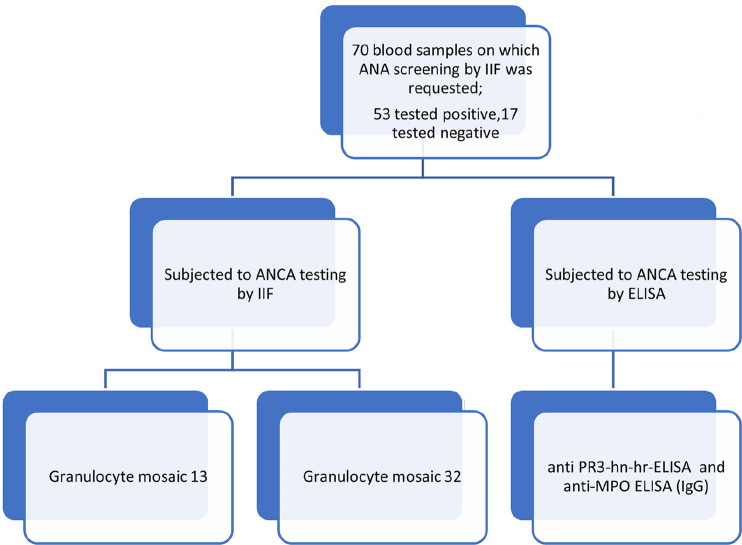
**(below).** Flowchart for selection of participants in the study.

**Figure 3.**
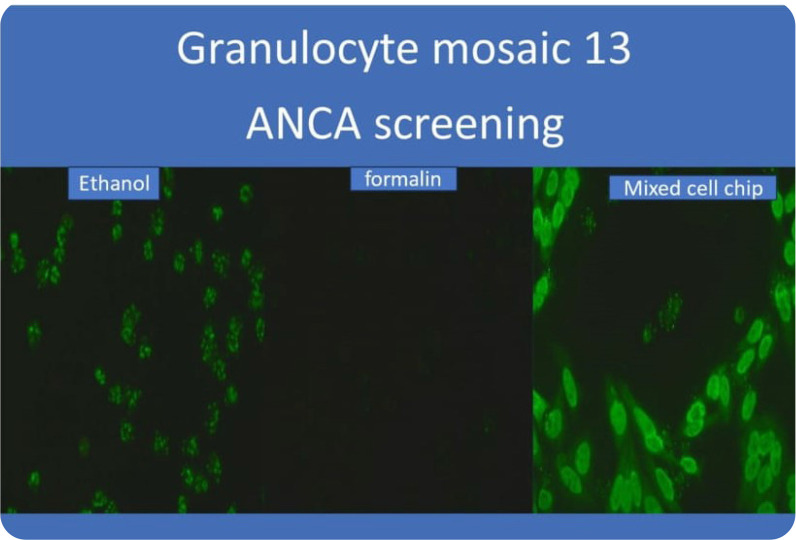
Three-biochip combination of granulocyte mosaic 13: Ethanol-fixed granulocytes, formalin fixed granulocytes, mixed cell chip (Hep-2 & EOH granulocytes).

**Figure 4.**
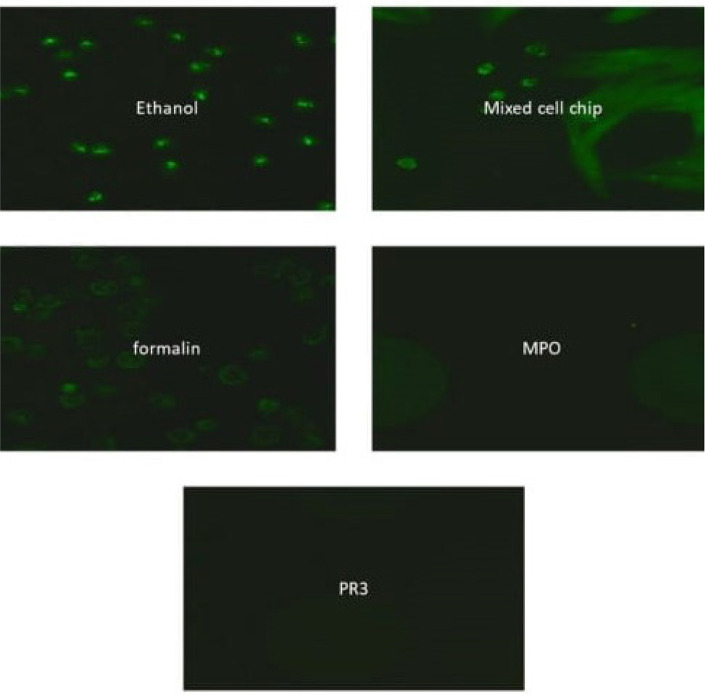
Granulocyte mosaic 32 showing five biochip combination: Ethanol-fixed granulocytes, formalin-fixed granulocytes, mixed cell chip (Hep-2 & EOH granulocytes), and MPO and PR3 dots.

Sera was separated after centrifugation and stored at −80° C deep freezer. ANCA analysis was performed on these 70 samples by three different methodologies: IIF Granulocyte mosaic 13 (Euroimmun, Lubeck, Germany), IIF Europlus Granulocyte mosaic 32 (Euroimmun, Lubeck, Germany) and ELISA for PR3 and MPO (anti PR3-hn-hr-ELISA IgG and anti-MPO ELISA IgG, Euroimmun, Lubeck, Germany) (**Figure 2**). ANA screening was performed using IIF mosaic: HEp -2010/Liver (Monkey) (Euroimmun, Lubeck, Germany). The approval for performing this research work was granted by Institutional Ethics committee, All India Institute of Medical Sciences, Patna via letter number AIIMS/Pat/IEC/2022/1042 dated 11/4/2023. Informed consent was taken from the participants. The ANA screening test was performed using IIF mosaic: HEp -2010/Liver (Monkey). Substrate was coated on each well on the slide in the form of HEp-2 epithelial cell/Primate liver biochip. Dilution of 1:100 was followed for ANA testing and processing was done via the automated sample processor Fluoromat 50 (CPC diagnostics). All samples for ANCA screening were processed via the automated Fluoromat 50 using patient sera diluted up to 1:10. Each biochip well on the Granulocyte mosaic 13 slide consisted of three biochips: ethanol fixed granulocytes, formalin fixed granulocytes and a mixture of HEp-2 cells and ethanol fixed granulocytes (mixed cell chip) (**Figure 3**). A supplementation of the classical ANCA screening test consisting of granulocyte substrates with the monospecific beads like MPO and PR3 was used to perform the ANCA profile test using IIF (dilution 1:10) Granulocyte mosaic 32 (**Figure 4**). It provides further diagnostic advantages like immediate confirmation in the same test run for positive results based on pattern identification. Clarity can also be obtained in case of unclear or non-specific fluorescence pattern. EUROS-tar LED microscope (Euroimmun) was used to read the slides by two different observers who were blinded to the ANA findings and results were recorded. All 70 samples were tested using monospecific anti-MPO and anti-PR3-hn-hr antibody ELISA kits (IgG ELISA, Euroimmun, Lubeck, Germany). The samples were analysed on the semi-automated ELISA analyser (Biotek).

### Analysis of IIF Results

**Figure 5.**
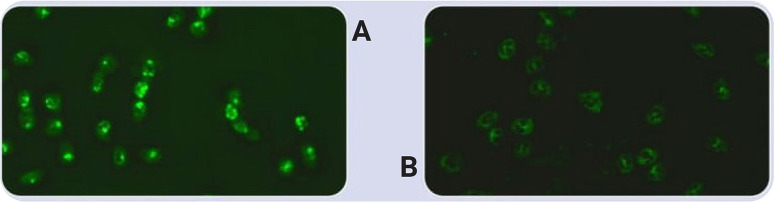
(**A**) pANCA appearance on ethanol-fixed granulocytes by IIFT. (**B**) cANCA appearance on ethanol-fixed granulocytes by IIFT.

The pattern and intensity level was determined and recorded for both ANA and ANCA. ANA patterns corresponding to homogenous, speckled, nucleolar, cytoplasmic and miscellaneous with an intensity of 2+ or more were included in our study. The ethanol fixed granulocytes and formalin fixed granulocytes were used to determine the pattern and intensity for ANCA by IIF. ANA positivity, pattern and intensity were evaluated using the mixed cell chip. The granulocyte mosaic 32 was supplemented with additional mono-specific antigens in the form of PR3 and MPO beads for further clarity when ambiguous ANCA results were observed based on patterns alone. ANCA patterns were analysed and categorised as cANCA, pANCA (**Figure 5A–B**) atypical pANCA, atypical inconclusive ANCA, and false positive pANCA.

### Statistical analysis

The primary aim of this study was to assess the concordance of ANCA reporting across three testing methodologies and to investigate the influence of ANA positivity on ANCA detection across three different platforms. We explored the impact of ANA positivity on ANCA reporting using logistic regression analysis where the dependent variable was ANCA positivity and the independent variables included ANA patterns, testing platforms and their interaction. An evaluation of the sensitivity and specificity of each ANCA testing platform was done considering ELISA as the reference method. Subgroup analyses were conducted on the basis of ANA patterns to further know their impact on the specificity and sensitivity of ANCA tests. All statistical tests were two- sided with a significance level set at 0.05. The statistical software JAMOVI 2.3.28 was used for the statistical analysis.^10^

## RESULTS

Chi square analysis was performed to evaluate the impact of ANA positive (n=53) and negative samples (n=17) on ANCA screening (IIF Granulocyte mosaic 13), ANCA profile (IIF granulocyte mosaic 32) and ELISA using PR3 and MPO antigens. p value was non-significant in all the three cases. **Table 1** reveals the impact of ANA positivity on ANCA reporting across different ANCA screening modalities namely IIF based granulocyte mosaic 13 and granulocyte mosaic 32 and ELISA using monospecific antigens PR3 and MPO.

**Table 1.** Comparative analysis of ANCA screening, ANCA profile, and ANCA ELISA profile test outcomes across overall ANA screening and ANA pattern wise.

	**ANCA Screening**			**ANCA Profile**			**ANCA ELISA Profile**		
**ANA screening**	Negative n (%)	Positive n (%)	Z-Test of Proportion	Negative n (%)	Positive n (%)	Z-Test of Proportion	Negative n (%)	Positive n (%)	Z-Test of Proportion
**Overall**									
Negative	76.5	23.5	0.211	82.4	17.6	0.498	82.4	17.6	0.227
Positive	88.7	11.3		88.7	11.3		92.5	7.5	
**Cytoplasmic**									
Negative	76.5	23.5	0.249	82.4	17.6	0.427	82.4	17.6	0.427
Positive	92.3	7.7		92.3	7.7		92.3	7.7	
**Homogenous**									
Negative	76.5	23.5	0.382	82.4	17.6	0.589	82.4	17.6	0.879
Positive	90	10		90	10		80	20	
**Speckled**									
Negative	76.5	23.5	0.711	82.4	17.6	0.456	90	10	0.589
Positive	70	30		70	30		85.2	14.8	
**Nucleolar**									
Negative	76.5	23.5	0.097	82.4	17.6	0.159	82.4	17.6	0.159
Positive	100	0		100	0		100	0	
**Miscellaneous**									
Negative	76.5	23.5	0.382	82.4	17.6	0.589	82.4	17.6	0.159
Positive	90	10		90	10		100	0	

Further it was intended to evaluate the impact of different ANA patterns on ANCA reporting using different methodologies. Significant p value was not obtained when chi square analysis was performed to evaluate the effect of cytoplasmic, homogenous, speckled, nucleolar, and miscellaneous ANA patterns versus negative ANA samples on ANCA screening using IIF granulocyte mosaic 13, ANCA profile using IIF granulocyte mosaic 32 and ELISA using MPO/PR3. **Table 1** reveals the impact of different ANA patterns on ANCA reporting using three different platforms, i.e. IIF based granulocyte mosaic 13, granulocyte mosaic 32, and ELISA. Chi square analysis was applied to analyse the effect of ANA positivity and negativity on the interpretations based on different (five) biochips/components of IIF Granulocyte mosaic 32(ANCA profile) i.e., ethanol fixed granulocyte, formalin fixed granulocytes, mixed well chip, PR3 and MPO coated beads individually. A significant p value was noted (p<0.001) when assessing the effect of ANA positivity on ANCA interpretation based on ethanol fixed granulocytes. However, a non-significant p value was obtained when chi square test was performed to look for any significant difference between ANA positivity and negativity on ANCA interpretation based on formalin fixed granulocytes and PR3/MPO dots. When Chi square analysis was applied to see if any significant difference existed between different categories of ANA positive (based on pattern) and negative samples on ANCA interpretation using ethanol fixed granulocyte, formalin fixed granulocytes, mixed well chip, PR3, and MPO dots individually, it was found that ANA positive samples with a homogenous pattern, nucleolar and miscellaneous pattern versus ANA negative samples revealed a significant p value in case of ethanol fixed granulocytes but not formalin fixed granulocytes or dot profile. **Table 2** reveals impact of ANA positivity along with the effect of different ANA patterns on ANCA reporting across five different components of granulocyte mosaic 32.

**Table 2.** Comparative analysis of ANCA profile test outcomes based on different biochips across overall ANA screening and ANA pattern wise.

	**ANCA Profile (Ethanol)**			**ANCA Profile (Formalin)**			**ANCA Profile (Dots)**		
ANA screening	Negative n (%)	Positive n (%)	Z-Test of Proportion	Negative n (%)	Positive n (%)	Z-Test of Proportion	Negative n (%)	Positive n (%)	Z-Test of Proportion
**Overall**									
Negative	64.7	35.3	<0.001	82.4	17.6	0.498	82.4	17.6	0.124
Positive	20.8	79.2		88.7	11.3		94.3	5.7	
**Cytoplasmic**									
Negative	64.7	35.3	0.153	82.4	17.6	0.427	82.4	17.6	0.427
Positive	38.5	61.5		92.3	7.7		92.3	7.7	
**Homogenous**									
Negative	64.7	35.3	0.006	82.4	17.6	0.589	82.4	17.6	0.159
Positive	10	90		90	10		100	0	
**Speckled**									
Negative	64.7	35.3	0.081	82.4	17.6	0.456	82.4	17.6	0.879
Positive	30	70		70	30		80	20	
**Nucleolar**									
Negative	64.7	35.3	<0.001	82.4	17.6	0.159	82.4	17.6	0.159
Positive	0	100		100	0		100	0	
**Miscellaneous**									
Negative	64.7	35.3	0.025	82.4	17.6	0.589	82.4	17.6	0.159
Positive	20	80		90	10		100	0	

The agreement analysis conducted in our study aimed to evaluate the concordance among three different ANCA testing platforms across various ANA patterns. Our findings revealed a high degree of agreement between ANCA screening and ANCA profile tests across all ANA status categories, with kappa values indicating almost perfect agreement. This suggests that both methods are highly consistent in identifying ANCA patterns, irrespective of the ANA status of the samples. Specifically, the kappa value for the overall ANA status comparison between ANCA screening and ANCA profile was remarkably high, demonstrating their reliability in ANCA detection across a diverse range of clinical samples. When comparing the ANCA screening and ELISA methods, the agreement was substantial but showed a decreased compared to the ANCA screening versus ANCA profile comparison. This variation in agreement levels may reflect the inherent differences in testing methodologies of IIF and ELISA. Despite this, substantial agreement indicates that ELISA remains a valuable tool for ANCA detection, particularly when specific antigens like PR3 and MPO are of clinical interest.

The comparison between ANCA profile and ELISA methods also demonstrated substantial agreement, further supporting the utility of these tests in clinical diagnostics. However, the slightly lower kappa values compared to ANCA screening vs ANCA profile comparison suggest that each method may have unique advantages and limitations, depending on the clinical context and the specific antigens being targeted. Interestingly, the analysis also highlighted the impact of ANA positivity on the agreement between these testing platforms. While the agreement remained high across ANA positive and negative status, slight variations were observed. This underscores the importance of considering ANA status in the interpretation of ANCA test results, as ANA positivity may influence the specificity and sensitivity of ANCA detection. **Table 3** depicts the agreement between three different testing modalities in case of ANA positive and negative samples. Our study also aimed to comment on the diagnostic accuracy of ANCA screening (IIF granulocyte mosaic 13) and ANCA profile (IIF granulocyte mosaic 32) keeping ELISA PR3/MPO as the gold standard. The overall sensitivity of the kit used for ANCA screening was 85.71% and diagnostic specificity was 93.65%. The overall sensitivity of the kit used for ANCA profile was 71.43% and diagnostic specificity was 93.65%. The overall diagnostic accuracy was 93.65% for ANCA reporting using either granulocyte mosaic 13 or granulocyte mosaic 32 for ANCA detection. **Table 4** explores the diagnostic accuracy of each ANCA testing platform in cases of ANA positivity and negativity.

**Table 3.** Inter-test agreement analysis: Kappa values with 95% confidence intervals for ANCA screening, profile, and ELISA across ANA status categories.

	N	Kappa value with 95% CI
**Overall ANA Status**		
ANCA Screening vs ANCA Profile	70	0.939 (0.821, 1.057)
ANCA Screening vs ELISA	70	0.667 (0.393, 0.941)
ANCA Profile vs ELISA	70	0.577 (0.271, 0.883)
ANA Positive Status		
ANCA Screening vs ANCA Profile	53	1.000 (1.000, 1.000)
ANCA Screening vs ELISA	53	0.560 (0.170, 0.950)
ANCA Profile vs ELISA	53	0.560 (0.170, 0.950)
ANA Negative Status		
ANCA Screening vs ANCA Profile	17	0.821 (0.489, 1.153)
ANCA Screening vs ELISA	17	0.821 (0.489, 1.153)
ANCA Profile vs ELISA	17	0.595 (0.081, 1.109)

The 95% CI for each Kappa value was calculated using the provided formula, where the lower limit is the Kappa value minus 1.96 times the Asymp. Std. Error, and the upper limit is the Kappa value plus 1.96 times the Asymp. Std. Error.

**Table 4.** Comparative diagnostic performance of ANCA screening and ANCA profile tests with 95% confidence intervals.

	**ANCA Screening**		**ANCA Profile**	
**Overall**		**95% CI**		**95% CI**
Sensitivity	85.71%	42.13% to 99.64%	71.43	29.04% to 96.33%
Specificity	93.65%	84.53% to 98.24%	93.65%	84.53% to 98.24%
Positive LR	13.5	4.99 to 36.53	11.25	3.91 to 32.40%
Negative LR	0.15	0.02 to 0.94	0.31	0.09 to 0.99
Diagnostic Accuracy	93.65	85.16 to 98.08%	93.65%	85.16 to 98.08%
**ANA Positive**				
Sensitivity	75%	19.41 %to 99.37%	75%	19.41 to 99.37%
Specificity	93.88%	83.13% to 98.72%	93.88%	83.13 to 98.72%
Positive LR	12.25	3.57 to 42.07	12.25	3.57 to 42.07
Negative LR	0.27	0.05 to 1.46	0.27	0.05 to 1.46
Diagnostic Accuracy	93.88%	83.70% to 98.61%	93.88%	83.70 to 98.61%
**ANA Negative**				
Sensitivity	100%	29.24 to 100	66.67%	9.43% to 99.16%
Specificity	92.86%	66.13 to 99.82	92.86%	66.13 to 99.82%
Positive LR	14	2.12 to 92.55%	9.33%	1.20 to 72.59
Negative LR	0	--	36.00%	0.07 to 1.79
Diagnostic Accuracy	92.86%	69.56% to 99.68%	92.86%	69.56% to 99.68%

ANCA: Anti neutrophil cytoplasmic antibody; ANA: Antinuclear antibody; CI: Confidence interval; LR: likelihood ratio.

## DISCUSSION

Distinct testing methodologies and platforms for ANCA commonly exhibit discrepancies in epitope and antigen expression and also in the detection of binding avidities leading to variation in results across different platforms.^11^ To address this variability in ANCA testing, standardisation of test methodology has been undertaken, notably utilising IIF on ethanol and formalin-fixed neutrophils, as described in the 1999 international consensus statement.^12^ However as per the recommendations of the Revised 2017 International Consensus, ELISA based methods replaced IIF as the primary autoantibody test in ANCA-associated vasculitis (AAV).^7^ However, this recommendation is contentious due to the robust performance of IIF ANCA and the inadequate standardisation of immunoassays for detection of antibodies to PR3/MPO. A revised document was published in 2020 as a follow up on 2017 recommendations on ANCA reporting which asserted the need for ANCA reporting in diseases beyond vasculitis.^8^ Therefore, when contemplating ANCA testing options, these limitations must be taken into account.

**Figure 6.**
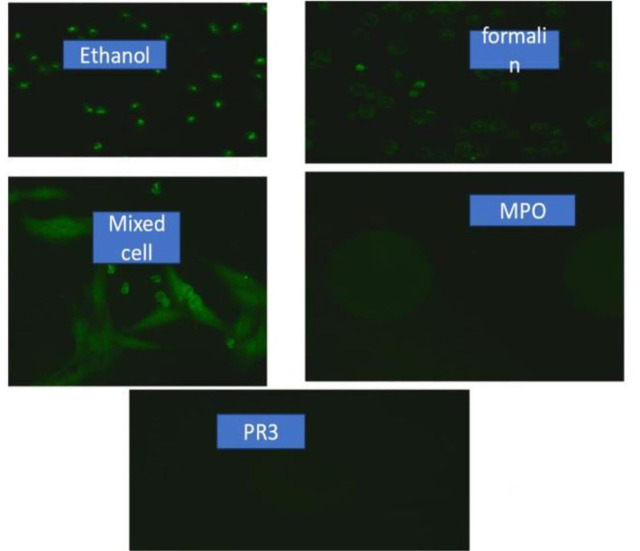
Ambiguous findings in granulocyte mosaic 32 dots (Both MPO and PR3 show positivity).

In view of unclear guidelines for ANCA testing, this study was undertaken to compare the results across the commonly existing testing methodologies for ANCA. This study reveals that all the three diagnostic platforms have considerable agreement as testing facility for ANCA detection with very good agreement amongst the two IIF based testing methodologies, i.e. Granulocyte mosaic 13 and granulocyte mosaic 32. The Granulocyte Mosaic 32 allows the simultaneous observation of both ethanol and formalin-fixed granulocytes ANCA IIF patterns along with the visualisation of monospecific MPO and PR3 microdots reactivity along with exclusion of ANA interference by use of mixed cell biochip (though a dilution of 1:10 should not be assumed as appropriate for ANA detection). In particular, any sample showing positivity for PR3 microdots with a corresponding cANCA pattern and a sample showing positivity for MPO microdots with a corresponding pANCA pattern should not need any further confirmatory testing. Similarly non-classical ANCA patterns such as formalin sensitive pANCA or atypical ANCA exhibited by particular samples, in combination with no reaction on the antigen-specific microdots, ideally do not need further testing in other MPO/PR3 mono specific ANCA immunoassays. This combination offers the benefit of single test versus multiple consecutive tests, so as to support early diagnosis, and hence, early treatment of AAV which is often needed to prevent significant morbidity and mortality. However, the good agreement between the two IIF based modalities in our study indicates no added advantage of the five-biochip well (IIF granulocyte mosaic 32) over the three-biochip wells (IIF granulocyte mosaic 13). The presence of MPO/PR3 dots did not seem to improve ANCA testing with respect to the granulocyte substrates comprising the three-biochip well. Practically observed differences were mainly due to ambiguous staining of the microdots with little or no fluorescence either alone or in combination. Scratches were often noted over the microdots and intervening area (**Figure 6**). Discrepancies also arose when the dots could not be visualised clearly due to a similar colour as the background. There is a possible need for further standardisation and validation of the MPO/PR3microdots in future studies. A study in past comparing the results of IIF and ELISA results showed a positive agreement in 124/129 (96.1%) samples and negative agreement in 540/542 (99.6%) samples.^13^ As the granulocyte mosaic 32 offered five biochips instead of the regular three offered in granulocyte mosaic 13, the financial implications rise with the use of the former with no added benefit as per results of our study. Our study reveals that this combination does not greatly facilitate the identification of ambiguous ANCA patterns on the basis of MPO or PR3 microdot reactivity. Moreover, the agreement was better between the two IIF based testing modalities in cases of ANA positive samples as compared to ANA negative samples. The 2016 and 2017 multicentre European Vasculitis Society (EUVAS) evaluation involving eight different antigen-specific immunoassays and four different IIF assays were evaluated. The AAV diagnostic accuracy for the IIF methods varied from 0.794 to 0.927, while the diagnostic accuracy of immunoassays varied from 0.922 to 0.954. This revealed good diagnostic performance of the antigen-specific immunoassays for ANCA detection in patients with MPA and GPA in contrast to high variability in perfor mance of IIF. The EUVAS study summarised that screening with IIF and follow-up testing with antigen-specific immunoassay was not a requisite for maximal diagnostic accuracy.^14,15^ These results suggested that the 1999 international consensus on ANCA testing for AAV required revision. In our study there was agreement between ANCA screening/ANCA profile and ELISA test methodologies, though not as strong as noted between the two IIF based technologies, probably due to intrinsic differences in nature of antigens involved. This agreement was on the lower side for ANA positive samples probably due to the phenomena of ANA interference on ANCA testing by IIF. However, the use of ELISA for an emergency test as ANCA may seem impractical, as ELISA is a batch test where an optimum number of accumulated samples justify a run, hence, further delaying the diagnosis. This becomes even more difficult in case of a rare disease like vasculitis. Moreover, ELISA may fail to identify any existing ANA positivity as the actual cause of symptoms in the presenting patient. IIF may provide a valuable clue in such a situation.

The diagnostic accuracy of both the IIF based kits was compared with reference to ELISA as the gold standard. The overall sensitivity and specificity for Granulocyte mosaic 13 was 85.71% and 93.61% respectively and the specificity and sensitivity for granulocyte mosaic 32 was 71.43 % and 93 .61%.As per a metanalysis by Guchelaar NAD, the pooled sensitivity estimate of cANCA by IIF was 75.2% (60.7% to 85.6%,95% CI) and the pooled specificity was 98.4% (92.8% to 99.7%, 95% CI), from a total of 9 studies conducted in the past. Four studies in total studied sensitivity and specificity for pANCA tested by IIF. The pooled sensitivity for pANCA is 46.3% (14.4% to 81.6%,95% CI) and the pooled specificity is 91.4% (80.8% to 96.4%, 95% CI).^16^

We also made an attempt to evaluate the impact of ANA positivity across different patterns on ANCA reporting across the three different platforms. None of the patterns were found to significantly impact ANCA reporting across three different platforms, i.e. Granulocyte mosaic 13, granulocyte mosaic 32, and ELISA. This is in contrast to earlier reported studies where presence of homogenous and mitotic spindle patterns have known to result in false positive pANCA reporting by IIF.^8^ Past studies have shown predominance of the false positive pANCA pattern by IIF in samples that tested positive for ANA due to the phenomena of ANA interference.^17^ The target proteins PR3 and MPO are both localised in the cytoplasmic granules of neutrophils and monocytes. They tend to be exposed on the cell surface due to diverse inflammatory stimuli. Ethanol fixation of neutrophils results in membrane solubilisation, causing migration of the target proteins from the granules. MPO, being more strongly cationic than PR3, redirects itself towards the nucleus with more negatively charged content, giving a perinuclear appearance. As ANA is also directed against nuclear antigens, a pANCA pattern is likely to be produced by some ANA pattern, mimicking presence of anti-MPO antibody. This also explains why the homogeneous pattern, which is often indicative of the presence of anti-double stranded DNA antibodies, is more frequently implicated in ANA interference.^18^ This interference, due to the process of binding to the substrate of proteins that rearrange themselves in the cytoplasm when the samples are fixed in ethanol, usually resolve upon formalin fixation as formalin does not cause membrane disruption, and hence, neutrophilic granules, MPO and PR3, remain in the cytoplasm.^19^ This phenomena poses challenges and hence a need for expertise in correct interpretation of ANCA pattern in cases of coexistence with ANA.

Having discussed the implications of using ethanol and formalin fixed granulocyte substrate together, we also tried to look for any significant differences between use of ethanol fixed granulocyte substrate alone or in association with formalin for ANCA reporting by IIF. Many studies in past have reported use of only ethanol fixed granulocyte substrate for ANCA reporting by IIF.^17,20^ Stone J et al. used both ethanol fixed granulocytes and formalin fixed granulocytes to explore the validity of IIF for ANCA diagnostics.^21^ The specificity increased from 76% to 93%, by use of formalin fixed granulocytes, as seen in the study by Hagen et al.^22^ In our study, the use of ethanol alone resulted in a significant number of ANCA false positives due to the existence of ANA in the same sample (p<0.001). The use of both ethanol and formalin substrates resulted in decline in false positive ANCA due to ANA interference. Use of ethanol alone resulted in significant false positives for ANCA by IIF in presence of homogenous, nucleolar, and miscellaneous ANA patterns. The false positive ANCA reported in case of homogenous ANA patterns was pANCA and cANCA in case of nucleolar ANA pattern. The addition of PR3/MPO dots added no significant advantage over ANCA reporting by ethanol and formalin combination. These results illustrate that with the use of multiple sub strates and fixatives simultaneously in a single-well IIF becomes a more reliable screening tool for ANCA. Conventional ANCA diagnosis using patient sera were based only on the visualisation of ethanol fixed granulocytes. Some laborato ries presently also use separate slides containing formalin fixed granulocytes to confirm ANCA positivity. The samples on formalin-fixed granulocytes are processed after obtaining positive pANCA results from ethanol-fixed granulocytes. This additional step is time-consuming, as the laboratory needs to wait for pANCA positive samples for the minimum strength of samples required for processing (3 or 5 usually), and is therefore impractical.

The sample size was limited with ANCA and ANA being rare parameters. Moreover, our study was confined to challenges faced in diagnostic tests used in the laboratory and not the clinical correlation/outcome.

Our study indicates the existence of ANA interference in ANCA testing by IIF while using only ethanol-fixed granulocyte substrates, which is reduced by the use of formalin fixed granulocytes. There is substantial agreement between the three testing methodologies for ANCA reporting, i.e. Granulocyte mosaic 13, granulocyte mosaic 32, and ELISA. The addition of PR3/MPO dots in granulocyte mosaic 32 over the three-biochip mosaic in granulocyte mosaic 13 provides no distinctive advantage in ANCA reporting in cases of ambiguous/inconclusive ANCA patterns by ethanol- and formalin-fixed substrates. Instead, it contributes towards added cost to the laboratory and patients. The use of re combinant PR3 (hr-PR3) from human source (HEK 293) as antigen in solid-phase in addition to human purified PR3 (hn-PR3) ensures optimum post translational processing and folding rendering conformational similarity to the native protein. This may offer better sensitivity as compared to first and second generation ELISAs, but this needs to be confirmed by further studies. Although monospecific assays are used in the detection of ANCA, IIF remains important and irreplaceable since many antigens are yet to be identified, and ANCA have a role beyond vasculitis, as in case of diseases like CIBD, infective endocarditis, autoimmune hepatitis, etc. This highlights that the indication for type of testing platform may also vary based on specialty. The study also emphasises that reflexing positive samples by any standardised methodology to either IIF or another antigen-specific immunoassay may not significantly improve diagnostic performance keeping in mind the good agreement between the different assays. This is in agreement with previously published work by Sepiashvili L et al.^23^ While the elimination of reflexive testing may ultimately facilitate decreased turnaround time in line with need for urgent reports and economical utilisation of resources, changing ANCA testing practices would require careful consideration and opinion of stakeholders.
